# Health-Related Quality of Life in Hemodialysis Patients: An Iranian Multi-Center Study

**DOI:** 10.5812/numonthly.12485

**Published:** 2013-08-18

**Authors:** Zohreh Rostami, Behzad Einollahi, Mahboob Lessan-Pezeshki, Azam Soleimani Najaf Abadi, Susan Mohammadi Kebar, Heshmatollah Shahbazian, Atieh Makhlough, Khadijeh Makhdoomi, Mahmood Salesi, Mojgan Jalalzadeh

**Affiliations:** 1 Nephrology and Urology Research Center, Baqiyatallah University of Medical Sciences, Tehran, IR Iran; 2 Department of Nephrology, Tehran University of Medical Sciences, Tehran, IR Iran; 3 Department of Nephrology, Artesh University of Medical Sciences, Tehran, IR Iran; 4 Department of Nephrology, Ardabil University of Medical Sciences, Ardabil, IR Iran; 5 Department of Nephrology, Ahvaz University of Medical Sciences, Ahvaz, IR Iran; 6 Department of Nephrology, Sari University of Medical Sciences, Sari, IR Iran; 7 Department of Nephrology, Urmia University of Medical Sciences, Urmia, IR Iran; 8 Department of Nephrology, Shahid Beheshti University of Medical Sciences, Tehran, IR Iran

**Keywords:** Quality of Life, Renal Dialysis, Mental Status Schedule, Kidney Diseases

## Abstract

**Background:**

The effectiveness of health care and health policy developments are often determined by health-related quality of life (HRQOL) assessment.

**Objectives:**

The objective of this study was to explore the potential corresponding factors and traditional biomarkers of HRQOL in a large number of Iranian hemodialysis patients.

**Patients and Methods:**

A total of 6,930 chronic hemodialysis (HD) patients enrolled. KDCS-SF version 1.3 questionnaire was used to assess the health related quality of life (HRQOL). We pooled PCS, MCS and KDCS scores with random effect model from 19 similar studies performed between 1996 and 2010

**Results:**

The mean age was 54.4 ± 17.1 years. Mean PCS, MCS and KDCS scores obtained for the study cohort were 40.79 ± 20.10, 47.79 ± 18.31 and 57.97 ± 11.70, respectively; the total score of SF-36 plus KDCS was 51.12 ± 13.41 as well. The most common primary known disease was hypertension (31.9%) and the second etiology was diabetes (25.5%). In multilevel logistic regression, Kt/V between 1 and 1.2 and PCS, KDCS more than 50 were considered as a significant reduction in the risk of hospitalization.

**Conclusions:**

This study showed that PCS and MCS score were slightly more than overall results while KDCS was slightly less than overall results. In addition, dialysis adequacy with Kt/V between 1 and 1.2 is associated with lower rate of hospitalization.

## 1. Background

Hemodialysis (HD) is a life-saving treatment for the patients with end-stage renal disease (ESRD) requiring renal replacement therapy (1). the health-related quality of life (HRQOL) is lessened in patients with ESRD as expected in those with chronic illness (2), because these patients have many fears and various necessities (3). Therefore, to achieve a high-quality life is a difficult issue, and needs a close cooperation between nephrologists, psychologists and social workers (4). The effectiveness of health care and development of health policies are often determined by HRQOL assessments (5). HRQOL is also an important predictor of HD patients outcomes that should be frequently assessed (6). The world health organization (WHO) characterized health as attendance of physical, mental, and social well-being and is not limited to the absence of disease (7); hence, the HRQOL measurement indicates the impact of illness on the patient’s physical, mental, and social performance (1).

Current studies (2, 7, 8) have publicized that a poor HRQOL in dialysis patients has strong correlation with increased rate of mortality. Thus, HRQOL is typically used to obtain information from patients, these information are not only focused on the health status, but also pointing out the risk of important outcomes such as death (8).

In HD patients, HRQOL may be affected by a number of elements, including clinical manifestations of disease, the treatment side effects, the quality of social activities, nutritional status, hospitalization (9), and some biochemical parameters such as Kt/V, calcium-phosphorus (Ca × P) product (1, 10), parathyroid hormone (PTH) levels (11, 12), anemia (9, 12-14), and serum albumin level (13, 15). It is important to note that different races, cultural diversity and various ethnicities may play role in HRQOL of dialysis patients (16), partly due to different perception of life and quality of their health care (17).

## 2. Objectives

The objective of this study was to explore the potential corresponding factors and traditional biomarkers of HRQOL in a large number of Iranian HD patients which can help to modify the health care strategies.

## 3. Patients and Methods

### 3.1. Patients

The Iranian adapted version of the kidney disease quality of life short form version 1.3 (KDCS-SF1.3) questionnaires was provided to all dialysis clinics in Iran with request for voluntary collaboration. A total of 6,930 chronic hemodialysis (HD) patients from 132 dialysis centers participated in this cross-sectional study, from October 2010 to August 2011. The patients 13 years old or older, with a clinically stable condition, with at least 3-month HD history, and receiving HD 3 times per week (each session lasts for 3 to 4 hours) were included in this study. The patients who were hospitalized for an acute illness, those with vascular access failure, including dialysis via a temporary vascular access and who refused to respond to the questionnaire were excluded.

### 3.2. Instruments 

KDCS-SF version 1.3 (18) questionnaire was used to assess health related quality of life (HRQOL). The KDCS-SF includes generic and disease specific cores. Generic core scales compromised of two components (mental and physical component summary, MCS and PCS), that including eight scales of the SF-36 physical functioning, role-physical, bodily pain, general health, vitality (energy/fatigue), social functioning, mental health (emotional well-being), and role-emotion. Disease specific items contain eleven scales targeted on kidney diseases and including symptoms/problems, effects of kidney disease on daily life, burden of kidney diseases, work status, cognitive function, quality of social interaction, sexual function, sleep, social support, dialysis staff encouragement, and patient satisfaction. These 11 kidney disease specific scales of KDCS-SF questionnaire validated in Iranian patients condensed as kidney disease component summary (KDCS). Each score is ranged from 0 to 100 point; so, higher scores indicated better life quality. The detailed translation and validation of SF-36 health survey have been described in another study (19); however, we have recently translated and validated KDCS-SF version 1.3 into Farsi (20). In this study, all scales internal consistency reliability measured according to Cronbach alphas ranging from 0.43 to 0.91 (Table 1). The questionnaire was generally self-administered, that the respondent fill out the form on his/her own; therefore, the patients mostly filled out their questionnaire at home or in dialysis department. The written information was double-checked with the patients to assure correct understanding and to make sure that they completed the questionnaire properly. 

Based on previous studies, in this analysis we used the cut-off scores of 43 for PCS (3); 51 (3) and 43 (2) for MCS; and 50 for KDCS as critical scores (4), below which the score is considered inadequate.

**Table 1.** Mean and Standard Deviation, Variability and Reliability of KDQOL-SF and SF-36 Items

	Mean ± SD	Cronbach’s alpha	Floor, %	Ceiling, %
**Symptoms**	67.9 ± 19.8	0.91	0.2	2.2
**Effects of kidney disease**	50.42 ± 20.9	0.79	1.4	0.6
**Burden of kidney disease**	23.08 ± 19.78	0.67	20.3	0.5
**Work status**	22.3 ± 34.56	0.43	62.3	11.3
**Cognitive function**	66.26 ± 21.21	0.63	2	8.01
**Quality of social interaction**	67.07 ± 20.08	0.49	0.2	7.1
**Sexual function**	63.48 ± 30.4	0.82	5.8	24.8
**Sleep **	55.9 ± 19.9	0.66	0.6	1.6
**Social support**	72.8 ± 26.9	0.78	3.04	34.3
**Dialysis staff encouragement**	81.3 ± 21.87	0.70	1.2	42.4
**Patient satisfaction**	69.01 ± 24.24	----	1.04	20.8
**Kidney disease component summary (KDCS)**	57.97 ± 11.7	0.68	0	0.01
**Physical function**	40.46 ± 29.5	0.92	9.05	2.6
**Rolephysical **	25.6 ± 32.7	0.74	51.4	8.7
**Pain**	55.31 ± 25.73	0.82	0.1	10.3
**General health**	41.70 ± 19.71	0.62	1.8	0.4
**Physical component summary (PCS)**	40.79 ± 20.1	0.71	0	0.04
**Emotional well-being **	54.24 ± 18.03	0.64	0.4	1.2
**Role emotional**	36.28 ± 38.89	0.73	2	0.8
**Social function **	55.77 ± 22.3	0.43	45.3	18.7
**Energy/ fatigue **	44.76 ± 19.79	0.62	1.3	7.1
**Mental component summary (MCS)**	47.79 ± 18.31	0.65	0.07	0.3
**SF-36**	44.29 ± 17.7	0.82	0	0.01
**SF-36 + KDCS**	51.12 ± 13.41	0.74	0	0.01

### 3.3. Data Analysis

Statistical analyses were performed using SPSS 18.0 for windows. Clinical, demographic, and HRQOL variables were expressed as means and standard deviations. Categorical variables were measured as frequencies and percentages. The one-way analysis of variance (ANOVA) or Kruskall-Wallis tests for skewed data used to compare continuous variables between more than two independent groups; the Student’s t-test or the Mann-Whitney test for skewed data was applied for comparisons between two groups. The Chi square test was used to compare categorical variables. Pearson correlation was used to assess the relationship between quality of life and continuous variables (e.g., age, dialysis vintage, Kt/V, and Hb). Following univariate analysis, all demographic and clinical variables with P ≤ 0.2 were entered as predictor variables in multiple regression models. A P-value of 0.05 or less was considered statistical significance.

We used two-level logistic regression, patients as level 1 and hospital as level 2, to estimate associations between hospital-level factor and hospitalization outcomes. We also pooled PCS, MCS and KDCS scores using a random effect model from 19 similar studies performed from 1996 to 2010 (Table 2). Then we compared our results with pooled data from all these studies. 

**Table 2.** Similar Researches Enrolled in Pooling Data

Reference	Year	Sample Size	Mean Age	M/F^a^ %	Country	PCS^a^	MCS^a^	KDCS^a^
(8)	2005 -2010	252	60.2 ± 15.5	65.9/35.1	Norwegian	36.6 ± 10.4	47.3 ± 11.0	
(21)	2011	202	52.5 ± 15.9	55.6/44.4	Brazil	42.2 ± 9.9	45.6 ± 14.6	
(5)	2006 -2007	223	69.5 ± 7.1	56.5/43.5	Brazil	38.28 ± 9.11	41.45 ± 9.88	69.27 ± 11.82
(22)	1995 -1998	949	57.7 ± 14.8	54.1/46.9	United States.	32.7 ± 10.0	46.6 ± 11.6	
(1)	2004 -2008	183	56.7 ± 15.9	56.3/44.7	Lithuania	40.6 ± 18.9	48.9 ± 19.7	
(23)	1997 - 2007	1010	63.2 ± 13.8	56.7/44.3	Netherlands	38.8 ± 9.7	43.7 ± 11.3	
(4)	2009	709	51.7 ± 12.6	54.7/46.3	Romania	46.3 ± 19.2	55.1 ± 19.3	68.3 ± 11.3
(10)	2006	27,154	61.5 ± 14.8	43/47	North America	33.2 ± 10.6	48.2 ± 11.2	
(24)	1995 - 2000	1,813	57.6	44/56	United States	35.75 ± 10.03	49.9 ± 10.89	
(25)	2006	71	59 ± 16	76/24	Denmark.	36.0 ± 10.2	51.1 ± 10.8	60.0 ± 19.8
(26)	1996 -2004	9,526	59.5 ± 14.8	58/42	DOPPS	35.7 ± 10.7	44.6 ± 11.9	
(27)	2004 -2005	112	55.5 ± 16.9	42/58	USA	36.2 ± 9.8	47.7 ± 11.0	62.2 ± 25.3
(28)	1996 -1997	1679	61.3	-------	United States	31.34 ± 9.7	44.32 ± 12.2	
(29)	2005	150	58.7 (18.21)	49.5/50.5	Irish	38.71 ± 10.08	51.12 ± 7.14	
(30)	1997	17,236	60.5 ± 15.2	57.4/43.6	DOPPS^a^	35.3_10.8	44.9 ± 11.9	63.5_13.0
(20)	2006 -2007	170	51.76 ± 18.37	58.9/42.1	Iran	39.4 ± 21.6	41.6 ± 20.9	52.6 ± 13.5
(31)	2009	100	53.4 ± 10.3	68.7/32.3	Saudi Arabia	52.7 ± 23.4	54.1 ± 24.5	59.7 ± 15.8
(32)	2011	78	52.35 ± 13.53	77/33	India	36.29 ± 12.25	33.29 ± 4.91	
Our study	2010 - 2011	6969	54.4 ± 17.13	56.8/44.2	Iran	40.79 ± 20.12	47.79 ± 18.31	57.97 ± 11.7

^a^ Abbreviations: DOPPS, dialysis outcomes and practice patterns study; M/F, male/female; KDCS, kidney disease component summary; MCS, mental component summary; PCS, physical component summary

## 4. Results

The demographic features of the 6930 patients who formed this study group and responded to the questionnaire are listed in Tables 3 and 4. The mean (+/-SD) age was 54.4 ± 17.1 years, with 1.3:1 male/female ratio, and a mean dialysis vintage of 37.5 ± 39.3 months. 

Mean PCS, MCS and KDCS scores obtained for the study cohort were 40.79 ± 20.10, 47.79 ± 18.31 and 57.97 ± 11.70, respectively; total score of SF-36 plus KDCS was 51.12 ± 13.41 as well. The most common primary known disease was hypertension and the second etiology was diabetes. Majority of patients were literate (53.4%) and married (72.7%). Only 10.1% of them were employed. Almost all patients were covered by insurance. The highest score for KDCS items was observed for dialysis staff encouragements (81.30 ± 21.87) and the lowest score was on work status (22.30 ± 34.56). The mean score of all domains, including SF-36 (8 items) and KDCS (11 items), was higher in male patients. The KDCS-SF (SF-36 and KDCS) domain scores and the laboratory data are shown in Table 1 and Table 4.

We found that 59% of our patients were anemic with hemoglobin level less than 10 g/dL for women and less than 11 g/dL for men; in 36.5% of cases the percent transferrin saturation (TSAT, i.e., = [serum iron ([micro]g/dL)/TIBC ([micro]g/dL)] × 100) was less than 20% and the serum ferritin level was less than 200 μg/L in 21% of subjects. Only 14% of patients were adequately dialyzed with Kt/V more than 1.2. Calcium-phosphorus (Ca × P) product less than 55 and PTH concentrations between 150 and 300 pg/mL were seen in 68% and 23% of subjects, respectively. Low serum albumin level (less than 4.0 g/dL) was observed in only 30% of patients; thus, the higher percentage of our dialysis patients had an adequate serum albumin level. Table 3 summarizes the mean level of all laboratory data. 

**Table 3.** Demographic and Laboratory Information

Variables	No. (%)
**Cause of ESRD, No. (%)**	
Hypertension	2109 (31.9)
Diabetes	1689 (25.5)
ADPKD	300 (4.5)
SLE	168 (2.5)
Infection	370 (5.6)
Others	774 (11.7)
Unknown	1202 (18.2)
**Educational State, No. (%)**	
Uneducated	2603 (47.6)
Primary school	1475 (27)
High school	1193 (21.8)
University	202 (3.7)
**Gender, No. (%)**	
Male	3897 (56.8)
Female	2961 (43.2)
**Age, No. (%)**	
≤ 45	1882 (28.6)
46 - 60	2089 (31.7)
> 60	2617 (39.7)
**Nationality, No. (%)**	
Iranian	6506 (96)
Non-Iranian	269 (4)
**Marital Status, No. (%)**	
Un-married	784 (11.7)
Married	4892 (72.7)
Widowed/Divorced	1051 (15.6)
**Work status, No. (%)**	
Employment	678 (10.1)
Unemployment	2622 (39)
Retired	1257 (18.7)
House keeper	2103 (31.2)
Student	71 (1.1)
**Age, y, mean ± SD**	54.4 ± 17.13
**Urea reduction ratio (URR), mean ± SD**	57 ± 0.14
**KT/V, mean ± SD**	0.9 ± 0.3
**Creatinine mg/dL, mean ± SD**	8.9 ± 3.3
**Sodiummmol/L, mean ± SD**	138.7 ± 4.59
**Potassiummmol/L, mean ± SD**	5.1 ± 0.81
**Calciummg/dL, mean ± SD**	9 ± 1.1
**Phosphorus mg/dL, mean ± SD**	5.6 ± 1.6
**Calcium phosphorus products, mean ± SD**	50.2 ± 15.9
**PTH pg/mL, mean ± SD**	417 ± 470 (10-3395)
**Alkaline phosphatas IU, mean ± SD**	379.65 ± 338.4 (10-5171)
**Hemoglubin g/dL, mean ± SD**	10.1 ± 1.8
**Serum ironμg/dL, mean ± SD**	90.1 ± 90.6
**Total iron binding capacity (TIBC) μg/dL, mean ± SD**	28.07 ± 17.07
**Transferin saturation (serum iron/TIBC) %, mean ± SD**	28.07 ± 17.07
**Ferritin ng/mL, mean ± SD**	747.65 ± 785.8 (320-5730)
**Triglyceride mg/dL, mean ± SD**	164.4 ± 98.9
**Cholesterol mg/dL, mean ± SD**	159.1 ± 45.06
**Low density lipoprotein (LDL) mg/dL, mean ± SD**	83.5 ± 30.6
**High density lipoprotein (HDL) mg/dL, mean ± SD**	38.7 ± 11.46
**Uric acid mg/dL, mean ± SD**	6.9 ± 1.8
**Albuming/dL, mean ± SD**	4.2 ± 0.64
**Aspartate aminotransferase (AST) IU/L, mean ± SD**	21.08 ± 23.05
**Alanine aminotransferase (ALT) IU/L, mean ± SD**	21.31 ± 30.85
**Prothrombin time second, mean ± SD**	14 ± 5.4

The elderly patients had significantly lower scores of all quality of life scales, except for social supports that no significant difference was observed. Staff encouragements was significantly better among middle-aged patients (45-60 years old) (P = 0.006) and patients' satisfaction was significantly increased with age (P < 0.001).

The higher educational level was significantly associated with better scores on all domains except the quality of social interactions that was abruptly decreased in patients with academic education (P ≤ 0.001). In addition, dialysis staff encouragement and patients' satisfaction decreased with high educational level (P = 0.002 and P ≤ 0.001, respectively).

### 4.1. PCS Score

In this study, the patients’ PCS average score was low (below 43) in 61 % of patients. Higher PCS score was significantly associated with non-anemic patients (41.84 ± 20.37 vs. 40.29 ± 19.77, P = 0.004), calcium-phosphorus (Ca × P) product less than 55 (41.34 ± 20.46 vs. 40.14 ± 19.2, P = 0.03), and male gender (42.5 ± 20.6 vs. 38.5 ± 19.1, P ≤ 0.001). There was a positive correlation between PCS score and serum albumin level (r = 0.12, P ≤ 0.001), serum creatinine concentration (r = 0.08, P ≤ 0.001), but weaker correlations with plasma sodium (r = 0.04, P = 0.001); on the other hand, a negative correlation was seen between PCS score and dialysis duration (r = -0.04, P = 0.001) and serum ferritin level (r = -0.064, P = 0.008).

After adjusting for covariates (including cause of disease, education, gender, age, hemoglobin, creatinine, plasma sodium, serum ferritin and albumin), only gender (B = 0.7, EXP B = 2.1, P ≤ 0.001) and age (B = -0.3, EXP B = 0.9, P ≤ 0.001) were the significant correlates of a PCS score more than 43.

### 4.2. MCS Score

The MCS score was less than 50 in 58.8% and less than 43 in 44.7% of patients. A significant association was seen between MCS score and males (48.65 ± 19.02 vs. 46.65 ± 17.26, P ≤ 0.001). In addition, a positive correlation was found between MCS score and hemoglobin level (r = 0.06, P ≤ 0.001), serum albumin value (r = 0.09, P ≤ 0.001), PTH concentration (r = 0.09, P = 0.01), serum creatinine level (r = 0.04, P ≤ 0.001), and plasma sodium (r = 0.04, P = 0.001); however, a negative correlation was observed between MCS score and serum ferritin level (r = -0.06, P = 0.004).

After adjustment for covariates (including the cause of disease, education, gender, age, sodium, PTH, serum ferritin, serum albumin and calcium-phosphorus (Ca × P) product), only age (B = -0.02, EXP B = 0.98, P = 0.04) and hemoglobin level (B = 0.1, EXP B = 1.1, P = 0.01) were significantly associated with MCS score of more than 43. In addition, hemoglobin level (B = 0.02, EXP B = 1.2, P = 0.007) was only associated with MCS score of more than 51, after adjustment for covariates including the disease cause , education, gender, age, sodium, PTH, serum ferritin, serum albumin and anemia.

### 4.4. KDCS Score

A KDCS score more than 50 was observed in 76.6% of participants. It was significantly associated with gender (male) (58.53 ± 12.07 vs. 57.21 ± 11.10, P ≤ 0.001). Moreover, hemoglobin level (r = 0.05, P ≤ 0.001), serum ferritin concentration (r = 0.1, P ≤ 0.001), serum albumin value (r = 0.06, P ≤ 0.001), PTH level (r = 0.09, P = 0.01), serum creatinine amount (r = 0.03, P = 0.01), and plasma sodium (r = 0.04, P = 0.001) had a significant correlation with KDCS, while a negative correlation was found between KDCS score and dialysis duration (r = -0.08, P ≤ 0.001).

After adjustment for covariates (including cause of disease, education, age, PTH, serum ferritin, serum albumin, dialysis duration, marital status and Kt/V), hemoglobin level (B = 0.02, EXP B = 1.2, P = 0.04) was only associated with KDCS score more than 50.

### 4.5. Outcome (Hospitalization)

The frequency of hospitalization was 50.2% (3504 patients) and median length of stay in hospital was 7 (1- 105) days. All scores of PCS, MCS and KDCS were higher in non-hospitalized patients compared to hospitalized patients (PCS, 38.64 ± 19.74 vs. 42.97 ± 20.27 P < 0.001; MCS, 46.10 ± 18.32 vs. 49.51 ± 18.14 P < 0.001; and KDCS, 57.22 ± 11.74 vs. 58.72 ± 11.72 P < 0.001).

Using one level regression analysis, Kt/V between 1 and 1.2 and PCS, MCS and KDCS more than 50 were significantly associated with lower rate of hospitalizations (Table 4). While in multilevel logistic regression, Kt/V ratio between 1 and 1.2 and PCS, KDCS more than 50 were associated with a significant reduction in the risk of hospitalization (Table 5). 

**Table 4.** One Level Regression for Hospitalization

Variable	OR	P-value	Random Effect (P-value)	Confidence Interval
**Calcium phosphorus products > 55 **	0.9	0.14	0.56	0.46-0.69
**PTH level 150-300 pg/mL**	0.84	0.5	0.92	0.5-1.5
**PTH level > 300 pg/mL**	0.89	0.62		
**Fasting blood glucose, mg/dL**	1.001	0.055	0.49	0.38-0.64
**Serum iron, μg/dL**	1.001	0.1	0.4	0.2-0.7
**Triglyceride, mg/dL**	1.0007	0.059	0.44	0.4-0.7
**Cholesterol, mg/dL**	1.0005	0.5	0.54	0.4-0.7
**Transferin saturation (Iron/TIBC) (%)**	0.99	0.25	0.41	0.24-0.7
**Ferritin level, ng/mL**	0.99	0.5	0.7	0.5-1.04
**Low density lipoprotein (LDL), mg/dL**	1.001	0.62	0.8	0.4-1.6
**High density lipoprotein (HDL), mg/dL**	0.99	0.52	0.8	0.4-1.6
**Uric acid > 6 mg/dL**	1.2	0.1	0.6	0.5-0.8
**KDCS** ^**a**^ ** > 50**	0.7	< 0.001	0.5	0.4-0.6
**PCS** ^**a**^	0.99	< 0.001	0.5	0.45-0.65
**MCS** ^**a**^	0.99	< 0.001	0.53	0.44-0.64
**PCS > 50**	0.7	< 0.001	0.5	0.45-0.65
**MCS > 50**	0.82	< 0.001	0.53	0.44-0.64
**SF-36 > 50**	0.76	< 0.001	0.53	0.44-0.64
**KDCS + SF-36 > 50**	0.7	< 0.001	0.53	0.44-0.64
**Hemoglobin level, g/dL**	1.007	0.6	0.57	0.46-0.7
**Age (y)**	0.99	0.8	0.51	0.42-0.62
**Anemia (Hemoglobin < 10 and < 11 for female and male respectively)**	0.95	0.4	0.54	0.46-0.7
**Kt/V 1-1.2**	0.69	< 0.001	0.46	0.33-0.63
**Kt/V > 1.2**	0.82	0.12		

^a^ Abbreviations: KDCS, kidney disease component summary; MCS, mental component summary; PCS, physical component summary

**Table 5.** Multilevel Regression Modeling for Hospitalization

	Mean ± SD	P Value	Relative Risk (Confidence Interval)
**PCS** ^**a**^ ** > 50**	0.7 ± 0.06	< 0.001	0.45 (0.32-0.62)
**KDCS** ^**a**^ ** > 50**	0.69 ± 0.07	0.001	
**Kt/V 1-1.2**	0.7 ± 0.07	0.001	
**Kt/V > 1.2**	0.83 ± 0.1	0.14	

^a^ Abbreviations: KDCS, kidney disease component summary; PCS, physical component summary

### 4.6. Pooled Results From Similar Studies

**Figure 1.**
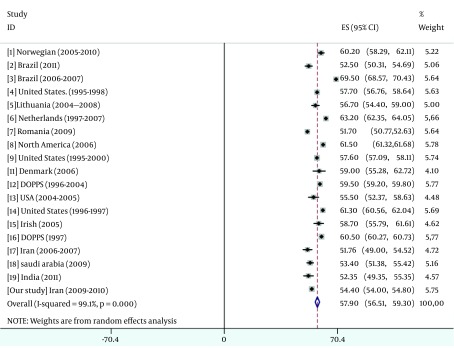
Overall Mean Age of Pooled Data

**Figure 2.**
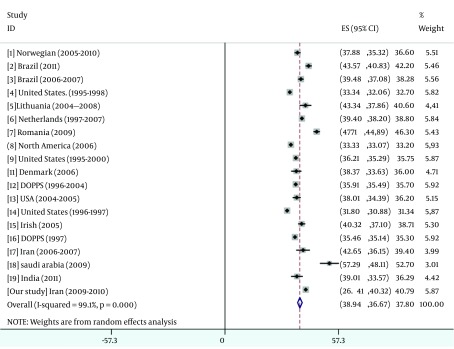
Overall Mean PCS Score From Pooled Data Overall Mean PCS Score From Pooled Data

**Figure 3 .**
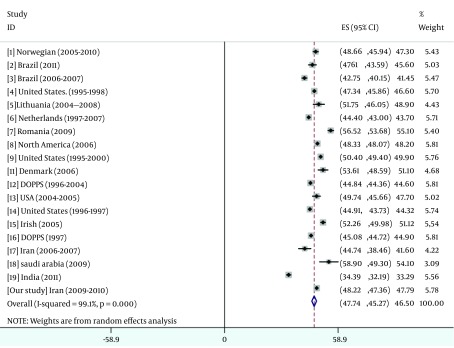
Overall Mean MCS Score From Pooled Data

**Figure 4.**
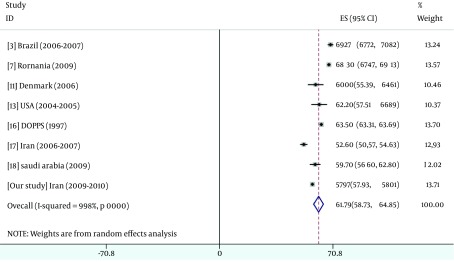
Overall Mean KDCS Score From Data

The result of pooled data were summarized in Figures 1, 2, 3 and 4.

## 5. Discussion

This national cross-sectional study shows the largest cohort of patients with ESRD on HD with QoL information reported from Iran. In the current study, we observed PCS and MCS scores were slightly higher than overall results that come from pooled data from 19 different studies; conversely, KDCS score was slightly lower than overall results. Based on several studies (25, 33, 34), Kt/V could not predict QoL in none of specific and non-specific components, we detected a U shape pattern for effect of Kt/V on hospitalization. On the other hand, the lowerrate of hospitalization was significantly observed in Kt/V between 1 and 1.2 and the amount lesser or more than this range associated with higher hospitalization possibility. We identified 3 potentially variables that represented the strongest association with hospitalization risk (PCS, KDCS and Kt/V ratio between 1 and 1.2).

According to pooling data, the patients in this study were younger than the patients in the 19 different studies (54.4 vs. 59.7). Since the prevalence of ESRD varies widely among different ethnic groups (35); for example, the south Asian population who live in United Kingdom and suffering from CKD has also a younger age than the indigenous white population (36). In addition, some risk factors of CKD such as diabetes and hypertension are more frequent in this population.

Although about 59% of the patients were anemic, the mean age was 54 years old. So better PCS and MCS scores might be expected, because they can tolerate some nonspecific aspects of physical and mental components, while reduced KDCS scores might be due to unsuitable state for dialysis adequacy predictors such as Kt/V, as almost only ¼ of our patients had 1 - 1.2 Kt/V and more than 60% had Kt/V less than 1. Significant improvement has been described in the survival of patients with optimized dialysis adequacy, despite the fact that few studies have found a correlation between the dialysis adequacy, Kt/V and urea reduction rate (URR) indices with HRQoL (33).

We conclude that hypertension and diabetes mellitus are the most common causes of ESRD among our HD patients. In this study, hypertension was the commonest cause of ESRD, followed by diabetes; however the majority of studies indicate diabetes mellitus as the first etiology of ESRD (24, 37, 38). The third most common category (18%) is the 'unknown origin', similar to the other studies, there was a significant number of ESRD patients with unknown cause; they usually developed with advanced renal failure and small size kidneys on renal ultrasonography; therefore, their disorders could not confirm with renal biopsy (36).

Although, evaluation of gender frequency among CKD patients, needs a systematic review and meta-analysis that is not aimed in our study, in contrast to HEMO study (24) but such as other studies (2, 5, 39) the prevalence of male patients were was higher than female. In addition, it may be considered that near all of KDCS scores were higher in male, as previously reported (4, 19, 20). It seems healthy behaviors and outlooks also vary among women and men (28). For example, based on previous studies some factors including perception of social support, religious conviction, and spirituality which persuade health outlook were reported more in women than men. Also questions dependent on physical strength may be scored with different values in both gender (28), and Hicks et al. (28) revealed different insight about health status and treatment preferences among women and men (28). On the other hand, Seica et al. believe that difference between male and female in HRQOL may be explained by women’s multiple domestic tasks and responsibilities that, unlike men, they cannot circumvent (4) such as housekeeping and breeding children. Furthermore, women never been retired.

In the present study similar to HEMO study (24), in univariate analysis only patients' satisfaction and dialysis staff encouragement were increased with the age. Although in the dialysis outcomes and practice patterns study (DOPPS), Lopes et al. (40) reported a low patient satisfaction score in Asian (Japanese) HD patients (66.7), our patients had slightly more satisfaction (69.01 ± 24.24). This difference could be due to cultural diversity between Iranian and Japanese patients.

Similar to other studies (5, 40-42), the worst dimension of quality of life was the occupational status, whereas in majority of them cognitive function and quality of social interaction were the best items.

In the current study, the effects of daily life, social support satisfaction, sleep, sexual function, staff encouragement and satisfaction were higher in women; however, similar to Vasquez et al. study (43), in multivariate level, gender had no effect on HRQOL.

Although a total score of HRQOL including MCS, PCS, and KDCS, and the majority of KDCS items (9/11) had a scale of 50 percent and more, but only ¼ of PCS and ½ of MCS items had more than 50 percent scale, which is relatively similar to Euro DOPPS and united state results (41). Moreover, we did not compare the results of SF- 36 with Iranian general population; low score for SF-36 indicating wide gap between these groups.

### 5.1. KDCS Score

Figure 3 revealed the mean KDCS score of our study was less than the results obtain from Romania (68.3) (4), Saudi Arabia (59.7) (42), DOPPS (61.7) (40), as well as US and Euro-DOPPS studies (63.7 and 62.7) (41). However, Mapes et al. (41) believe that KDCS may contributes to a more in-depth assessment of the HRQOL of HD patients, but the results suggest that it may be contributed to predictions of death and hospitalization when information about PCS and MCS is available and factors within the MCS and PCS can explain the associations between KDCS and outcomes. On the other hand, Lopes et al. (40) found that no significant difference was observed between Asians and Caucasian for the MCS and KDCS. 

### 5.2. MCS and PCS Scores

A few data are available concerning the quality of life among Iranian HD patients in general. Our study showed that the quality of life scores among Iranian HD patients were compared to other studies (Figures 2, 3 and 4). About 35% of our HD patients had both PCS and MCS scores lower than the critical scores that were established by Lowrie et al. (3). Similar to the previous studies, MCS score of our patients was higher than PCS score due to dynamic adaptation of patients to their chronic illness (4). The difference between MCS and PCS was almost the same (+7) that reported by Seica et al. (4) and was higher than (+4.5) Turkmen study (2) and Braga (+3.17) (5)and lower than Lacson et al. (+15) (10) study and the study conducted in Saudi Arabia (+1.5) (42).

### 5.3. Hospitalization

Similar to Zhang et al. study (44), half of our patients were at least one time hospitalized during this study. Spending time in hospital was similar to the mean length of stay in European countries which varied between 8.7 days in the UK to 14.7 days in Germany (median length of stay varied from 4 to 10 days for these countries) (45). Kalantar-Zadeh et al. (25) reported that prospective hospitalizations of hemodialysis patients significantly correlate with the SF-36 total score and its two main dimensions. Our study showed an inverse association between HRQOL and hospitalization for all three components of the KDQOL-SF (i.e., MCS, PCS, and KDCS) but after adjustment for several risk factors of hospitalization this associations statistically remained significant just for the PCS and KDCS scores more than 50. It is important to note that, according to the results of this study, the associations between hospitalization and dialysis adequacy had a U shape pattern (23).

### 5.4. Limitation

There are no HRQOL data were obtained in general population and CKD individuals in Iran. Therefore, we could not compare the quality of life in HD patients with the general population and CKD cases. The mortality rate of ESRD patients was unrevealed, so we could not evaluate the correlation between survival and HRQOL.

### 5.5. Conclusion

This study showed that PCS and MCS scores were slightly more than the overall results while KDCS was slightly less than the overall results. In addition, dialysis adequacy with Kt/V between 1 and 1.2 is associated with a lower rate of hospitalization.
